# Assessing Validity of Self-Reported Dietary Intake within a Mediterranean Diet Cluster Randomized Controlled Trial among US Firefighters

**DOI:** 10.3390/nu11092250

**Published:** 2019-09-19

**Authors:** Mercedes Sotos-Prieto, Costas Christophi, Alicen Black, Jeremy D Furtado, Yiqing Song, Prokopios Magiatis, Aikaterini Papakonstantinou, Eleni Melliou, Steven Moffatt, Stefanos N. Kales

**Affiliations:** 1 Department of Environmental Health, Harvard University T.H Chan School of Public Health, Boston, MA 02215, USA; costas.christophi@cut.ac.cy (C.C.); stefokali@aol.com (S.N.K.); 2 Division of Food and Nutrition Sciences, School of Applied Health Sciences and Wellness and Diabetes Institute, Ohio University, Athens, OH 45701, USA; ab914017@ohio.edu or; 3 Department of Preventive Medicine and Public Health, School of Medicine, Universidad Autónoma de Madrid, C/Arzobispo Morcillo, 28029 Madrid, Spain; 4 Department of Nutrition, Harvard T.H Chan School, Boston, MA 02115, USA; jfurtado@hsph.harvard.edu; 5 Cyprus International Institute for Environmental and Public Health, Cyprus University of Technology, 30 Archbishop Kyprianou Str., Lemesos 3036, Cyprus; 6 Department of Epidemiology Indiana University Richard M. Fairbanks School of Public Health, Indianapolis, IN 46202, USA; yiqsong@iu.edu; 7 Department of Pharmacy, National and Kapodistrian University of Athens, Panepistimiopolis Zografou 157 71, Greecekath.papakonstantinou@gmail.com (A.P.); emelliou@pharm.uoa.gr (E.M.); 8 National Institute for Public Safety Health, Indianapolis, IN 324 E New York Street, Indianapolis, IN 46204, USA; steven.moffatt@ascension.org

**Keywords:** mediterranean diet, biomarkers, validation, compliance

## Abstract

Collecting dietary intake data is associated with challenges due to the subjective nature of self–administered instruments. Biomarkers may objectively estimate the consumption of specific dietary items or help assess compliance in dietary intervention studies. Our aim was to use a panel of plasma and urine biomarkers to assess the validity of self-reported dietary intake using a modified Mediterranean Diet Scale (mMDS) among firefighters participating in Feeding America’s Bravest (FAB), an MD cluster-randomized controlled trial. In our nested biomarker pilot study, participants were randomly selected from both the MD intervention group (*n* = 24) and the control group (*n* = 24) after 12-months of dietary intervention. At baseline data collection for the pilot study (t = 12-months of FAB), participants in the control group crossed-over to receive the MD intervention (active intervention) for 6-months. Participants in the intervention group continued in a self-sustained continuation phase (SSP) of the intervention. Food frequency questionnaires (FFQ), 13-item-mMDS questionnaires, 40 plasma fatty acids, inflammatory biomarkers and urinary hydroxytyrosol and tyrosol were analyzed at both time points. Spearman’s correlation, *t*-tests and linear regression coefficients were calculated using SAS software. Overall, the mMDS derived from the FFQ was highly correlated with the specific 13-domain-mMDS (r = 0.74). The concordance between the two questionnaires for low and high adherence to MD was high for all the participants in the parent trial (κ = 0.76). After 6 months of intervention in the pilot study, plasma saturated fatty acid decreased in both groups (active intervention: −1.3 ± 1.7; *p* = 0.002; SSP: −1.12 ± 1.90; *p* = 0.014) and oleic acid improved in the SSP (*p* = 0.013). Intake of olive oil was positively associated with plasma omega-3 (*p* = 0.004) and negatively with TNF-α (*p* < 0.001) at baseline. Choosing olive oil as a type of fat was also associated with higher levels of plasma omega-3 (*p* = 0.019) at baseline and lower TNF-α (*p* = 0.023) at follow up. Intake of red and processed meats were associated with lower serum omega-3 (*p* = 0.04) and fish consumption was associated with lower IL-6 at baseline (*p* = 0.022). The overall mMDS was associated with an increase in plasma omega-3 (*p* = 0.021). Good correlation was found between nutrient intake from the FFQ and the corresponding plasma biomarkers (omega-3, EPA and DHA). In this MD randomized controlled trial, some key plasma biomarkers were significantly associated with key MD diet components and the overall mMDS supporting the validity of the mMDS questionnaire as well as compliance with the intervention.

## 1. Introduction

Collecting dietary intake data using dietary assessments, such as food frequency questionnaires (FFQ) and diet scores derived from nutrition and lifestyle questionnaires, provides researchers with a general understanding of dietary habits. However, due to the self-reported nature of these methods, data obtained through these subjective assessments are prone to bias [1]. Subjective assessments are sensitive to human error, including misreported information and recall bias that may potentially skew research findings [2]. To overcome some of the limitations of the use of subjective nutritional assessment, other objective methods are often used to compliment data collection. Objective methods are quantifiable and less prone to error when compared to subjective assessments and, therefore, are able to provide more accurate information. In nutrition assessment, biomarkers are often used in conjunction with subjective methods to assess the consumption of specific foods, food groups or nutrients of interest and to help assess compliance in dietary intervention studies. Additionally, biomarkers are used to determine the reliability and validity of subjective dietary assessments and can help document compliance in dietary intervention studies [3,4,5]. In contrast some drawbacks to the used of biomarkers are cost, participant discomfort and time.

Several studies have assessed Mediterranean Diet (MD) compliance with the use of diet scores, however, the majority of these scores were validated in older, Mediterranean populations [6]. Limited research exists regarding the MD’s effects on middle aged, occupationally active, non-Mediterranean populations and there are currently no validated methods to assess MD compliance in this population.

Feeding America’s Bravest is a cluster-randomized-controlled trial that aimed to assess the efficacy of an MD intervention among the 44 fire stations of the Indianapolis Fire Department [7,8]. To that end, a modified Mediterranean Diet Score (mMDS) to measure MD adherence [6] had been developed in a similar Midwestern firefighter population. A previous cross-sectional study in these firefighters using the mMDS found that participants in the highest quartile of mMDS compared to the lowest, had a 35% lower risk for the presence of an additional metabolic syndrome component. Additionally, higher HDL-cholesterol and lower LDL-cholesterol were observed in those with higher mMDS adherence [6]. However, while the cross-sectional results in the above-mentioned study among firefighters indirectly support the validity of the mMDS, there were two limitations: (1) the validity of the mMDS questionnaire had not yet been assessed against validated questionnaires and (2) the score is unable to biologically estimate the consumption of specific key MD dietary components. Mono and polyunsaturated fats from sources such as olive oil, nuts and fish, including oleic acid, omega-3 and omega-6, have been observed to change in response to MD consumption [5,9,10,11,12,13,14,15]. Additionally, elevated consumption of olive oil has been previously associated with urinary tyrosol and hydroxytyrosol [16,17]. These bioactive compounds, consumed as part of the MD, have been shown to positively affect the inflammatory response markers, such as Tumor Necrosis Factor (TNF-α), Interleukine-6 (IL-6) and C-Reactive Protein (CRP) [18,19,20]. By validating the mMDS within the current intervention is important because a successful MD intervention would suggest that our approach could be disseminated among other public safety professions or workplaces in non-Mediterranean populations. Therefore, the aim of the current pilot study was (1) to assess the validity of the mMDS questionnaire with a validated FFQ and the use of biomarkers and (2) to use a panel of plasma biomarkers of fatty acids (40 fatty acids, including oleic acid, omega 3 and omega 6) and systemic inflammation (CRP, IL-6 and TNF-α), in addition to urine biomarkers (tyrosol and hydroxytyrosol) to assess compliance with an MD intervention among a random sub-sample of firefighters participating in the parent trial, “Feeding America’s Bravest.”

## 2. Materials and Methods

### 2.1. Study Design

**Figure 1:**
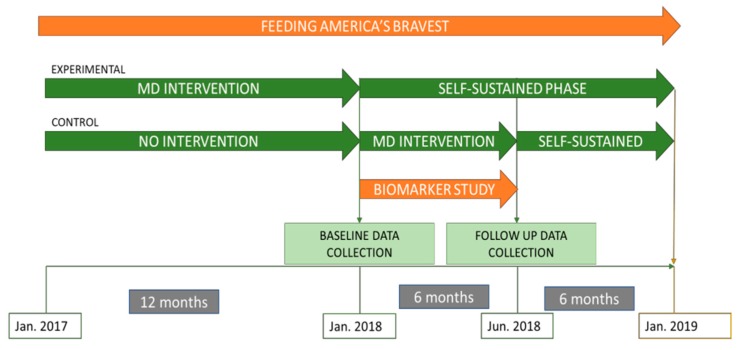
Study design and timeline of “Feeding America’s Bravest” Mediterranean diet nutrition intervention study. January 2018 marks the beginning of the current pilot biomarker sub-study.

“Feeding America’s Bravest” is a cluster-randomized controlled trial among the 44 fire stations of the Indianapolis Fire Department. Briefly, the aim of the study was to compare a Mediterranean nutrition educational intervention (experimental group) to an ad-libitum, Midwestern-style diet (“usual care” control group) for 12-months, followed by a cross-over in which the experimental group underwent a self-sustained continuation phase of the intervention for an additional 12-months to determine the extent and persistence of resulting behavior change [7]. The control group at 12-months crossed-over to receive an active Mediterranean Diet Nutritional Intervention for another 6-months to test the efficacy of the same but shorter, intervention followed by 6-months of a self-sustained phase (SSP) (Figure 1). For this pilot study we randomly selected a subgroup of participants (*n* = 48) whose fire stations had been assigned to the MD intervention (*n* = 24) or the control group (*n* = 24) for the initial 12-month period (i.e., at the 12-month follow-up of the parent study *Feeding America’s Bravest*). At the time of recruitment into our sub-study, the participants in the control group crossed-over to the intervention for another 6 months (“active intervention”) and participants in the intervention stayed in a SSP. The current study evaluated lifestyle habits and biomarkers at two time points (baseline [12 months of the parent study] and after 6 months [18-months of the parent study]). (Figure 1)

### 2.2. Study Population

Participants from “Feeding America’s Bravest” were contacted at random via e-mail to participate in the current pilot study. They were informed that they would receive incentives (including a gift card for participation). Recruitment proceeded down the list of randomly selected parent-study participants until 24 had volunteered from the intervention group and 24 from the control group. The inclusion and exclusion criteria are summarized as follows: Indianapolis Fire Department (IFD) members eligible for participation included those that were permanently assigned to one of the 44 IFD stations, had a fire department-provided medical exam within the two years prior to recruitment, were at least 18 years of age and were either full duty, modified or restricted duty status at the time of consent or were categorized as “Administrative Staff” by the IFD. IFD members not eligible for participation included those without a recorded fire department exam in the two years prior to recruitment [7].

### 2.3. Diet Assessment

Dietary intake and lifestyle assessments were completed using a validated FFQ in addition to a lifestyle questionnaire that included a 13-item mMDS, questions on physical activity and other questions regarding lifestyle habits, such as sleep patterns [6,21,22,23,24]. Both questionnaires were completed at baseline of the current study and again after 6-months. Using the 13-domain mMDS questionnaire, adherence to the MD was calculated for each participant according to a scoring system, as previously described by Yang et al. and modified by the inclusion of two additional domains (nut and legume consumption) (Supplementary Table S1) [6]. For each question a scale of up to 4 or 5 points was created where the minimum score of 0 represents a choice that least conforms to the MD and the maximum score represents a choice that most conforms to the MD. The dietary domains, along with their respective point scales, include the consumption of fast food (0–4), fruits (0–4), vegetables (0–4), legumes (0–4), nuts (0–4), sweet desserts (0–4), fried foods (0–4), ocean fish (0–4), breads and starches (consumed at home and the fire station (0–4)), the type (0–2) and frequency of alcoholic beverages (0–4), non-alcoholic beverages (consumed at home and the fire station (0–4)) and the type of cooking oil or fat (consumed at home and the fire station (0–5)). For domains evaluated in both the homes and fire stations of the participants (breads and starches, non-alcoholic beverages and oils/fats), the percentage of meals consumed at each location was considered in order to compute the weighted score for each item. The mMDS was calculated by summing the scores across all domains, with the possible range of the total mMDS score being from 0 (no conformity to the MD) to 51 (maximum conformity to the MD) (Supplementary Table S1) Dietary information was also collected using a validated, 131-item semi-quantitative FFQ [24]. Participants were asked how often, on average, they consumed each food of a standard portion size in the previous year. The responses ranged from “never or less than once per month” to “six or more times per day” [24,25,26]. The mMDS was derived from the FFQ to assess the agreement with the short-screener 13-domain mMDS. The FFQ was assessed for the overall participants of Feeding America’s Bravest at baseline (*n* = 420) and in the pilot study at the two time points, baseline (12 months of the parent study] and after 6 months [18-months of the parent study]). We then evaluated the validity of the mMDS at all three time points. Briefly, the PREDIMED score consists of 14-questions of which 12 questions are about food consumption frequency and 2 questions on food intake habits considered characteristic of the Spanish Mediterranean diet. Each question was scored 0 or 1, with a total possible range of 0 to 14 (higher scores indicate greater adherence to the MedDiet).

### 2.4. Biomarker Assessment

Blood samples were collected after an overnight fast at baseline and at follow-up. Using EDTA collection tubes up to 15 mL of blood was collected. Plasma and serum were aliquoted, frozen at −80 °C, stored and run in batches. Fatty acids and inflammatory biomarkers were analyzed at the Nutritional Biomarker Laboratory at the Harvard T.H. Chan School of Public Health and the remaining plasma and blood samples were stored for future studies. Forty fatty acids, including oleic, alpha-linolenic and alpha-linoleic acids, all of which are reliable indicators of the consumption of monounsaturated fatty acids (MUFAs), tree nuts and other healthy fats, were measured by gas chromatography, as described previously [27,28]. Groups of fatty acids based on its composition were created (e.g., omega 3 fatty acids included Alpha Linolenic acid, Eicosapentaenoic acid, docosapentaenoic acid and docosahexaenoic acid.) Briefly, fatty acids were extracted and prepared for analysis by transmethylation using methanol and sulfuric acid. Following esterification, the fatty acid methyl esters were suspended in iso-octane and quantitated by gas-liquid chromatography as fully described by Baylin et al. [9]. Two duplicate control samples were included in each batch and across all batches to allow for calculation of within and between batch coefficients of variation. Quality control was maintained by external validation through participation in programs offered by both the American Oil Chemists Society and the National Institute of Standards and Technology.

Inflammatory biomarkers were assessed using commercially available ELISA kits (Cat# HS600B Human IL-6 Quantikine HS ELISA Kit; Cat# HSTA00E Human TNF-alpha Quantikine HS ELISA Kit; Cat# DCRP00 Human C-Reactive Protein/CRP Quantikine ELISA Kit; R&D Systems, Minneapolis, MN). All samples were analyzed in duplicate and any pairs with a coefficient of variation >20% were repeated.

Urine samples were collected at the same time as blood samples. First spot morning urine was obtained by participants at home, following an overnight fast, according to specific instructions. Hydroxytyrosol and tyrosol (known biomarkers for olive oil consumption and main metabolites of olive oil phenolic ingredients that are found in urine) were measured after hydrolysis with hydrochloric acid, extraction and derivatization with MSTFA, using gas chromatography–mass spectrometry coupled to a mass spectrometer detector system Separation of Tyrosol and Hydroxytyrosol was conducted using an HP-5 MS (DB-5) (30 m × 0.25 mm i.d. and 0.25 μm film thickness) cross-linked 5% phenylmethylsilane capillary column at the National and Kapodistrian University of Athens, Greece. The analytic procedure has been published previously [29].

### 2.5. Statistical Analysis

Quantitative characteristics as continuous variables are presented as mean ± SD and compared between the two groups using the independent *t*-test. Categorical characteristics are shown as sample size (frequency) and differences between groups were assessed using the chi-square test of independence. Spearman’s correlation coefficients were calculated to quantify the agreement of being above or below the median value between the short-screener 13-item mMDS and the corresponding mMDS derived from the FFQ in the overall population, as well as in the pilot study both at baseline and at the 6-months follow-up. The agreement between the two scores, was evaluated further with the use of the Cohen’s kappa coefficient and the intra-class correlation (ICC), together with the corresponding 95% confidence intervals. The ICC was calculated as the difference of the between subjects variation and the within subjects variation, as resulting from a one-way ANOVA, divided by the sum of the two variations. 95% CIs were computed assuming a normal distribution. We used the icc_sas macro as provided in Reference [30]. In addition, Bland-Altman plots are presented showing the relation between the mean difference of the two indices obtained from the FFQ and the corresponding mMDS questionnaires with the corresponding mean of the two indices. In addition, the PREDIMED score was calculated, a previously validated MD score to evaluate MD adherence [31], to assess the correlation with the mMDS.

Furthermore, paired *t*-tests were used to evaluate whether there was a significant change in the biomarkers of interest from baseline to 6-months in either the intervention or the control group and *t*-tests were further utilized to assess whether these mean changes were different between the two groups. Linear regression models, adjusted for group, were performed to quantify the effect of different modified Mediterranean Diet food groups and the overall mMDS on dietary biomarkers in the blood and urine.

All analyses were done using the statistical software SAS v9.4 (SAS Inc., Cary, NC). All tests performed were two-sided and the statistical significance level was set at *p* < 0.05.

## 3. Results

### 3.1. Participants Characteristics

Baseline characteristics of the overall participants (*n* = 420) in the Feeding America’s Bravest study and for the biomarkers pilot study (*n* = 48) are presented in Table 1 overall and by treatment group. There were no significant differences in age, body-mass index (BMI), smoking, gender or the overall mMDS by groups at baseline in either the overall study or the biomarkers pilot study except for BMI in the latter. Most of the participants were male in both the parent study and the pilot study (94.7% and 92.8% respectively) with few females participating in the pilot study (7.3%). Race differed significantly in the parent study between treatment groups, with Caucasians more prevalent in the control group (89.6%) compared to the intervention group (76.6%). No significant differences were observed in biomarker values at baseline in the pilot study.

### 3.2. Modified Mediterranean Diet Score Agreement

The absolute and relative agreements of mMDS between the FFQ and the mMDS questionnaire were calculated for the overall sample in the Feeding America’s Bravest as well as at baseline and at 6-month for the biomarkers pilot study (Table 2).

The concordance for low and high mMDS was highest for the overall population at baseline for FAB (κ = 0.76 and ICC = 0.76). At baseline for FAB, most of the participants were correctly classified as low or high adherence to MD (88% total) based on mMDS under both methods evident at 6-months in the pilot study (Table 2).

**Figure 2:**
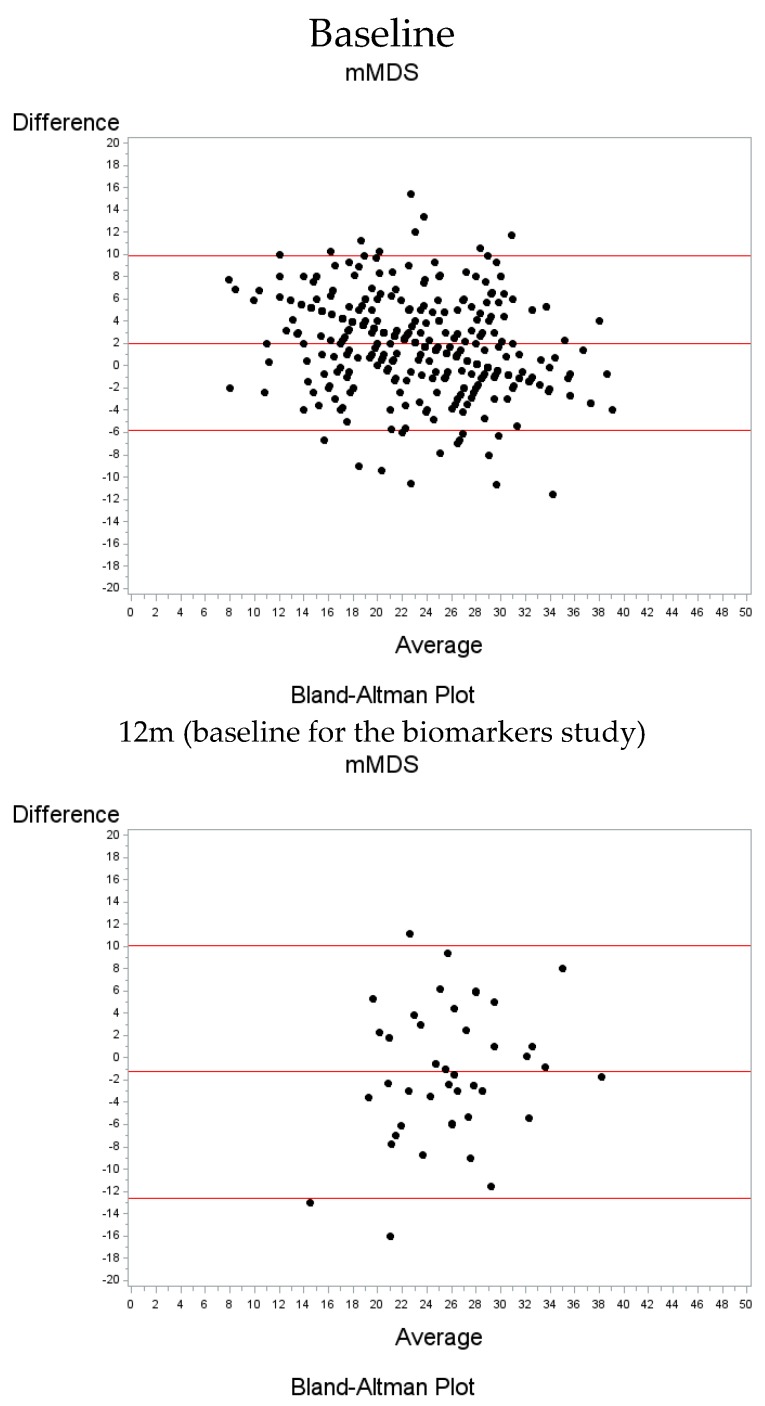

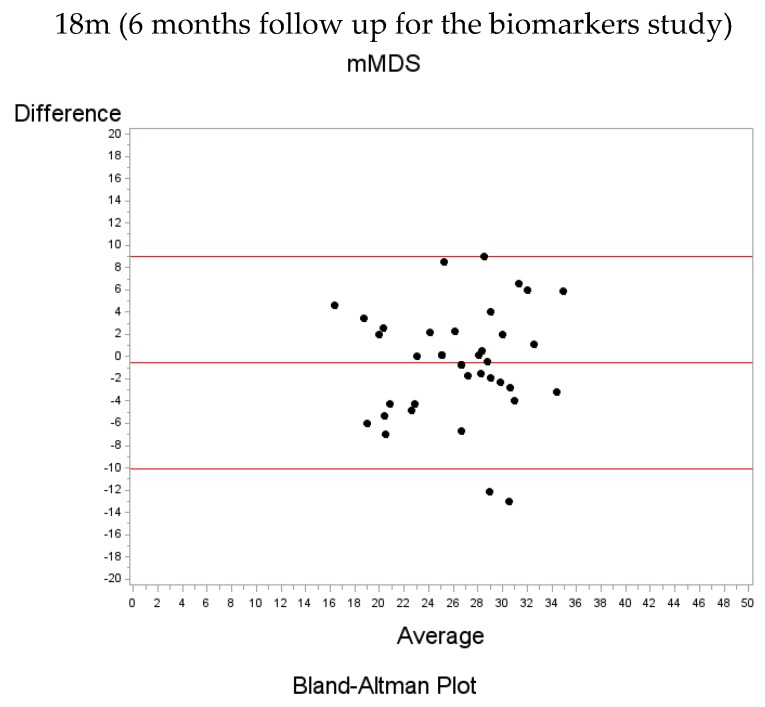
Bland-Altman Plots indicating the mean difference between indices obtained from the FFQ and the corresponding mMDS questionnaires vs the mean of the two indices for the overall population [baseline] and the pilot study [12 months follow up (baseline for the pilot study); 18 months follow up (6 months follow-up for the pilot study)]. The red lines refer to the mean difference as well as the lower and upper bounds (2 standard deviations above and below the mean). The dots correspond to the different participants.

At baseline, the mMDS questionnaire was on average 2.02 units higher than the corresponding mMDS derived from the FFQ value, 1.26 units lower at 12 months and 0.56 units lower at 18 months (Figure 2). The majority of the observations are within 2 standard deviations of the mean difference and no particular trend is observed as the average of the scores increases.

The overall correlation between the mMDS-derived FFQ and mMDS scores were high (overall FAB r = 0.74, baseline pilot study (12-months FAB) r = 0.42 and follow-up (6 months pilot study and 18 months FAB) r = 0.59) (Table S2). In addition, correlations with another validated MD score (the PREDIMED score) was calculated and these were strong as well (r = 0.72) (Table S2). Looking at individual items that comprise the score revealed that the highest correlations were between type of oil or fat used more often (r = 0.50), weekly consumption of fried food (r = −0.42) and type of alcohol consumed (r = 0.50). The lowest correlations were seen for servings of fruits per day (r = 0.04), type of beverages consumed with most meals (r = −0.06), consumption of bread and starches with meals (r = 0.16) and serving of nuts per week (r = 0.16)

### 3.3. Plasma and Urine Biomarkers Assessment

Plasma and urine biomarkers were measured in the pilot study at both baseline and 6-months. After 6 months of follow-up, there was a decrease in saturated fatty acids in both, the self-sustained continuation phase (mean change −1.12 ± 1.89; *p* = 0.014) and the group in the active intervention (−1.35 ± 1.68; *p* = 0.02) and an increase in oleic acid in the self-sustained continuation phase (mean change 1.05 ± 1.78; *p* = 0.013) compared to baseline (Table 3). No significant differences were found for urine biomarkers.

The association between each item of the mMDS and the overall mMDS with plasma and urine biomarkers after adjusting for the group assigned was also analyzed (Table 4). There was a significant and positive linear association of olive oil consumed with plasma omega-3 (β = 0.66; R^2^ = 0.24; *p* = 0.004) and an inverse association with TNF-α (β = −0.26; R^2^ = 0.31; *p* < 0.001) at baseline. Choosing olive oil as the type of fat was also associated with higher levels of plasma omega 3 (β=0.89; R^2^ = 0.18; *p* = 0.019) and lower TNF-α at follow-up (β = −0.37; R^2^ = 0.15; *p* = 0.023). In addition, consumption of red and processed meats was associated with lower omega-3 (*p* = 0.046) at baseline, fish consumption with lower IL-6 at baseline (*p* = 0.022) and alcoholic beverages with hydroxytyrosol at follow-up (*p* = 0.009). The overall mMDS was associated with an increase in plasma omega-3 (*p* = 0.021).

Table 5 outlines the correlation between nutrient intake from the FFQ and the corresponding plasma fatty acid biomarkers. There were consistent and significant positive correlations of dietary intakes of omega-3 fatty acids and docosahexaenoic acid (DHA) with the same plasma biomarkers at baseline (omega-3: r = 0.624; *p* = 0.001; DHA: r = 0.673, *p* < 0.001) and after the active intervention (n-3: r = 0.741; *p* <0.001; DHA: r = 0.775, *p* < 0.001). Eicosapentaenoic acid (EPA) from food was also positively associated with the same plasma biomarker at baseline and after 6 months in both groups.

## 4. Discussion

In the current study, we evaluated the validity of the 13-domain mMDS questionnaire to assess adherence to an MD pattern among firefighters against two high standard instruments: a previously validated, 131-item FFQ and a battery of dietary biomarkers. Regardless of either high or low adherence to the MD, the agreement between the mMDS questionnaire and the mMDS-derived from FFQ showed excellent concordance (>0.75) [32,33] among the overall population and in the pilot follow-up study. Additionally, more than 80% of participants’ MD pattern (high or low adherence to MD) were correctly classified by both questionnaires. These findings are similar to previous research that has found comparable results between the ability of full-length FFQs and shorter questionnaires to assess, identify and classify MD dietary adherence [34,35,36].

By food item, strong correlations were found for olive oil as type of oil used, consumption of fried foods and the type of alcohol ingested. The lowest correlations were found with fruit intake, the consumption of bread and starches with meals and the type of beverage consumed with meals. Similar correlations between food items have been reported in other studies [35,36,37]. While the mMDS questionnaire measures food habits directly with straightforward responses (e.g., how many servings of nuts do you consume per week?), the FFQ assesses dietary consumption more generally and with at least 9 different responses (e.g., how often on average have you consumed 1 oz. of walnuts during the past year?). In addition, in the FFQ, food items are evaluated individually, using standardized portion sizes and are then calculated to estimate the overall consumption of specific food groups over the previous 12-months. Due to differences in specificity and format, this could mean that the mMDS questionnaire is more sensitive for evaluating MD adherence than the FFQ. It has been shown that dietary patterns are reported with more accuracy when asked in multiple, individual questions (like in the mMDS), as opposed to single, grouped questions [38]. Additionally, accuracy has been shown to increase when questionnaires address the consumption of different types of foods (e.g., milk; whole, skim, 1%, 2%, etc.) using nesting methods as opposed to multiple separate questions (as in in the FFQ) [38].

To further assess if adherence to MD could be measured by the mMDS, the association between key components of the MD and select biomarkers was analyzed. Traditionally, the MD is known for its high consumption of fat (>40%), specifically mono and polyunsaturated fatty acids (MUFA and PUFA) from primarily olive oil, nuts and fish sources. We found that choosing olive oil (over other sources of fat) and greater consumption of olive oil was associated with higher levels of plasma omega-3, a PUFA. Although olive oil is characterized by its high content of oleic acid (omega-9), a MUFA, previous research has shown that higher consumption of olive oil has been associated with changes in concentrations of omega-3 [36,39] despite changes in concentrations of omega-9 [39]. Similar to previous research, we did not find an association between choosing or consuming olive oil and omega-9 levels. However, levels of omega 3 improved with MD intervention in the self-sustained continuation group compared to baseline, supporting compliance to the intervention.

Consumption of olive oil was also associated with lower TNF-α at baseline. TNF-α is an inflammatory cytokine that has been linked with obesity, T2DM, hypertriglyceridemia, decreased HDL-cholesterol and CVD, among other inflammatory and infectious diseases. When elevated, TNF-α has been shown to correlate to the severity of disease and also acts as a predictor of CVD and T2DM. However, scientific literature has observed that only olive oil supplemented within the context of the MD has been shown to influence TNF-α levels [40,41,42]. Olive oil is an antioxidant rich and MUFA dense and intake of only 50 mL per day, in addition to the consumption of an MD, has shown significant reductions of inflammatory biomarkers, including TNF-α. But when supplemented in the habitual non-MD diets of overweight, obese and diabetic subjects TNF-α production remained unaffected [40,41,42]. This suggests that the addition of olive oil, outside of an MD context, may not elicit the same anti-inflammatory effects. High in fruits, vegetables, nuts and legumes, the effects of the MD on inflammation may be enhanced by olive oil consumption as it is well known that the intake of oils increases the bioavailability of polyphenols and carotenoids [40,41,42]. Additionally, factors such as BMI, body composition, disease status, physical activity level, microbiome, behavioral and genetic factors all influence the effects of olive oil consumption in reducing TNF-α [42]. As can be seen in our data (Table 1), our participants had a high prevalence of obesity.

In regard to tyrosol and hydroxytyrosol in response to olive oil intake, no statistically significant associations were found. Immediate and extended olive oil consumption has previously been associated with strong dose responses to both phenolic compounds [16,17]; however, in these studies participants were required to consume at least 25mL of olive oil per day; whereas Feeding America’s Bravest participants were only encouraged to increase olive oil consumption. It is also possible that the consumption of alcoholic beverages like beer or wine that contain also significant quantities of tyrosol may have masked the effect of olive oil [43].

Additionally, our study found that the consumption of red and processed meat was associated with lower omega-3 levels. Contrary to previous research, we did not find a statistical association between meat consumption and omega-6 [44]. Exogenous in nature, omega-3 and omega-6 fatty acids are not produced in the body and can only be obtained through dietary consumption, therefore, circulating concentrations of PUFA act as reliable indicators of their consumption [5,13,15]. Low-fat dietary patterns are associated with decreased plasma concentrations of both omega-3 and omega-6 [45]. However, meat consumption correlates with elevated plasma levels of arachidonic acid [44], an omega-6 PUFA found predominantly in grain-fed animals, dairy and eggs [46]. After 6-months of follow-up, there was a 4–5% decrease in SFA plasma biomarkers in both intervention groups. Because SFAs can be altered endogenously, plasma SFA concentrations are not considered reliable indicators of saturated fat intake [11,47]. It has been observed that in carbohydrate restricted diets, where the primary source of fat came from unsaturated fatty acid sources, plasma SFA decreased while carb restricted diets, where the primary source of fat came from SFA sources, saw no changes in plasma SFA [48].

In agreement with other studies [36,49], we found that the consumption of at least 3 servings of fish per week was associated with higher plasma omega-3 levels, with no changes in omega-6 levels. Fish consumption was also associated with lower IL-6 levels at baseline. IL-6, another inflammatory cytokine, has previously been inversely associated with MD consumption. In their study, Mena et al. found that when assessed in response to a low-fat dietary intervention, IL-6 levels remained static or even increased, while IL-6 levels in the MD group showed consistent, significant decreases [50]. Fish consumption has also been shown to decrease biomarkers of low grade inflammation (including IL-6) even after being adjusted for sex, energy intake, BMI, physical activity, alcohol consumption, smoking behavior and other food groups (including fruits, vegetables and dairy) [51].

Omega-3 was associated with, individual components of the MD as well as with the overall mMDS. Diet quality scores such as the Alternate Healthy Eating Index [52,53], Brazilian Healthy Eating Index [54] and the Diet Quality Index [55] have also been positively associated with omega-3 levels. This raises the question of whether omega-3 could potentially be validated as a biomarker for diet quality.

In addition, in our study specific self-reported nutrients evaluated from the FFQ showed a strong correlation with the corresponding biomarkers in plasma both at baseline and after 6-months of the intervention. Specifically, this was true for omega-3, EPA and DHA. In contrast, palmitoleic acid, a known SFA, showed a negative correlation supporting the validity of the self-reported diet. The additional correlation between PREDIMED score and mMDS supports that the mMDS is capturing the MedDiet pattern.

This study is limited by its small sample size and the possibility of some volunteer bias among the randomly selected firefighters who declined or accepted to participate in the biomarker study. In addition, the results of this study may not be generalizable to other working populations or females, as the study population for the biomarkers pilot study was majority male. Furthermore, while many biomarkers were analyzed in this study, we did not measure all relevant biomarkers, only a selection. Lastly, the Feeding America’s Bravest parent study did not initially call for the use of biomarkers for validation and, therefore, true baseline data (typically collected before the start of a dietary intervention) could not be assessed. Future research should aim to conduct a larger, more comprehensive biomarker study.

## 5. Conclusions

In conclusion, in this MD cluster-randomized controlled trial, we showed the good agreement of mMDS scores between the 13-item mMDS questionnaire and the mMDS derived from a previously validated 131-item FFQ and also found that some key plasma biomarkers were significantly associated with key MD diet components and the overall mMDS. Overall, our findings support the validity of the mMDS questionnaire in characterizing habitual MD pattern and assessing compliance of MD intervention in such an occupationally active, non-Mediterranean population of US firefighters. With this study we intend to contribute knowledge regarding the use of nutritional biomarkers for dietary assessment and objective measures of compliance in MD intervention studies, specifically within workplace interventions among people from non-Mediterranean origins, which have been studied infrequently.

## Figures and Tables

**Table 1:** Baseline demographic and lifestyle characteristics of the parent trial and pilot trial.

	Overall Feeding America’s Bravest				Pilot –Biomarkers Study (Baseline)			
	All (*n* = 420)	Control (*n* = 214)	Intervention (*n* = 206)	*p*-Value	All (*n* = 48)	Control (*n* = 24)	Intervention (*n* = 24)	*p*-Value
Age (years)	48.36 ± 8.29	49.02 ± 7.86	47.67 ± 8.68	0.096	47.52 ± 7.63	47.58 ± 8.63	47.46 ± 6.67	0.955
Gender				0.140				0.520
Males	250 (94.7%)	122 (96.8%)	128 (92.8%)		38 (92.7%)	20 (95.2%)	18 (90.0%)	
Females	14 (5.3%)	4 (3.2%)	10 (7.2%)		3 (7.3%)	1 (4.8%)	2 (10.0%)	
Race				0.003				0.563
Caucasian	217 (82.8%)	112 (89.6%)	105 (76.6%)		31 (79.5%)	16 (80.0%)	15 (78.9%)	
African American	39 (14.9%)	9 (7.2%)	30 (21.9%)		7 (17.9%)	3 (15.0%)	4 (21.1%)	
Other	6 (2.3%)	4 (3.2%)	2 (1.5%)		1 (2.6%)	1 (5.0%)	0 (0.0%)	
BMI (kg/m^2^)	29.97 ± 4.48	30.13 ± 4.47	29.80 ± 4.50	0.447	29.68 ± 3.50	31.13 ± 3.07	28.24 ± 3.35	0.003
BMI group				0.785				0.094
18.5–25	49 (11.9%)	24 (11.4%)	25 (12.4%)		5 (10.4%)	1 (4.2%)	4 (16.7%)	
25–30	185 (44.8%)	98 (46.4%)	87 (43.1%)		20 (41.7%)	8 (33.3%)	12 (50.0%)	
30+	179 (43.3%)	89 (42.2%)	90 (44.6%)		23 (47.9%)	15 (62.5%)	8 (33.3%)	
Smoking				0.433				0.520
Yes	11 (4.2%)	4 (3.1%)	7 (5.1%)		3 (7.3%)	1 (4.8%)	2 (10.0%)	
No	254 (95.8%)	123 (96.9%)	131 (94.9%)		38 (92.7%)	20 (95.2%)	18 (90.0%)	
Overall mMDS (0–51 points)	24.08 ± 5.73	24.38 ± 5.61	23.78 ± 5.85	0.284	25.02 ± 5.79	26.00 ± 5.00	24.04 ± 6.44	0.245
FFQ-derived mMDS	22.05 ± 6.89	21.95 ± 6.97	22.16 ± 6.82	0.753	26.42 ± 4.79	27.88 ± 4.78	24.96 ± 4.44	0.034
CRP (ngmL)	N/A	N/A	N/A		1733 ± 2041	1640 ± 1936	1827 ± 2179	0.754
TNF-α (pgmL)	N/A	N/A	N/A		1.09 ± 0.35	1.14 ± 0.39	1.04 ± 0.31	0.308
IL-6 (pgmL)	N/A	N/A	N/A		1.92 ± 2.15	1.69 ± 1.14	2.15 ± 2.84	0.489
MUFA (%)	N/A	N/A	N/A		22.33 ± 3.16	22.98 ± 3.16	21.69 ± 3.08	0.157
PUFA (%)	N/A	N/A	N/A		47.14 ± 4.15	46.89 ± 4.00	47.39 ± 4.37	0.682

**Table 2:** Agreement between the modified Mediterranean Diet Score (mMDS) and the mMDS derived from the food frequency questionnaire (FFQ) in the overall population at baseline and in the lot study at both baseline and 6 months follow-up.

	mMDS FFQ Derived						
mMDS Questionnaire	Agree (Low-Low)	Agree (High-High)	Disagree (Low-High)	Disagree (High-Low)	r	k (95%CI)	ICC
Overall (parent trial baseline)	183 (43%)	192 (45%)	27 (6%)	24 (6%)	0.76 (*p* < 0.001)	0.76 (95% CI: 0.70, 0.82)	0.76 (95% CI: 0.72, 0.80)
Pilot study baseline	15 (31%)	15 (31%)	8 (17%)	10 (21%)	0.25 (*p* = 0.08)	0.25 (95% CI: −0.02, 0.52)	0.11 (95% CI: −0.21, 0.41)
Pilot study 6 m follow-up	15 (37%)	19 (46%)	4 (10%)	3 (7%)	0.66 (*p* < 0.001)	0.66 (95% CI: 0.42, 0.89)	0.65 (95% CI: 0.44, 0.79)

**Table 3:** Levels and changes in plasma, urine biomarkers and mMDS at baseline and 6 months follow up in the pilot trial of “Feeding Americas Bravest.”

Variable	Control Active Intervention										Intervention Self Sustained-Continuation Phase										*p* ^
	Baseline			6-Months Follow-Up			Changes			*p* #	Baseline			6-Months Follow-Up			Changes			*p* #	
	N	Mean	SD	N	Mean	SD	N	Mean	SD		N	Mean	SD	N	Mean	SD	N	Mean	SD		
TNF-α (pgmL)	24	1.14	0.39	20	1.22	0.44	20	0.053	0.228	0.315	24	1.04	0.31	21	1.21	0.35	21	0.181	0.289	0.009	0.123
IL6 (pgmL)	22	1.69	1.14	20	1.88	2.48	18	0.209	2.668	0.744	22	2.15	2.84	21	2.51	4.29	20	0.363	5.279	0.762	0.909
CRP (ngmL)	24	1640	1936	20	1084	1232	20	−619	1656	0.111	24	1827	2179	21	1584	1783	21	−19	2185	0.969	0.330
Tyrosol (ppm)	18	0.019	0.026	14	0.024	0.015	9	0.013	0.019	0.072	19	0.020	0.033	16	0.016	0.006	13	0.002	0.014	0.569	0.149
Hydroxytyrosol (ppm)	23	0.105	0.094	17	0.090	0.082	16	−0.018	0.082	0.400	24	0.137	0.105	17	0.095	0.073	17	−0.033	0.120	0.275	0.681
SFA (%)	24	29.01	1.42	20	27.64	1.46	20	−1.347	1.679	**0.002**	24	29.74	1.66	21	28.49	1.80	21	−1.118	1.897	**0.014**	0.686
Oleic Acid (%)	24	19.45	3.06	20	19.64	2.08	20	−0.042	2.897	0.949	24	18.02	2.83	21	19.06	2.81	21	1.052	1.766	**0.013**	0.157
Alpha-linolenic Acid (%)	24	31.36	3.37	20	32.69	3.33	20	1.461	3.682	0.092	24	31.60	3.83	21	31.10	3.88	21	−0.756	3.917	0.387	0.070
Linoleic Acid (%)	24	0.63	0.23	20	0.68	0.26	20	0.023	0.219	0.645	24	0.64	0.16	21	0.67	0.17	21	0.004	0.210	0.938	0.775
Omega 3 fatty acid (%)	24	3.35	0.91	20	3.54	0.96	20	0.056	0.553	0.654	24	3.91	1.25	21	3.82	1.08	21	−0.082	0.978	0.704	0.579
mMDS (0–51 points)	24	26.00	5.00	22	26.93	4.74	22	1.063	4.613	0.292	24	24.04	6.44	22	25.05	5.47	22	1.231	5.140	0.274	0.909

TNF-α: Tumor necrosis factor; IL-6: Interleukin 6; CRP: C-Reactive Protein; SFA: Saturated Fatty Acids; mMDS: modified Mediterranean Diet Score; FFQ: Food frequency questionnaire. # paired *t*-test testing whether the mean difference is different than 0 ^ *t*-test testing whether the mean difference in Group 0 is different than the mean difference in Group 1.

**Table 4:** Modified Mediterranean Diet food groups (from the mMDS questionnaire) association with plasma and urine biomarkers at baseline and 6 months follow-up in the pilot study of the Feeding Americas Bravest.

	Baseline (*n* = 48)				6 Months Follow-Up (*n* = 41)			
	β	SE	*p*	R^2^	β	SE	*p*	R^2^
Olive oil with								
CRP (ngmL)	−582.06	402.82	0.157	0.10	676.12	365.72	0.077	0.13
TNF-α (pgmL)	−0.257	0.066	<0.001	0.31	−0.012	0.114	0.919	0.002
IL-6 (pgmL)	−0.156	0.543	0.776	0.03	0.653	0.611	0.296	0.069
MUFA (%)	−0.518	0.623	0.412	0.08	0.123	0.648	0.852	0.004
Oleic acid (%)	−0.437	0.586	0.460	0.08	0.124	0.545	0.822	0.009
Omega 3 (%)	0.663	0.218	0.004	0.24	0.491	0.272	0.083	0.120
Omega 6 (%)	−0.162	0.850	0.850	0.00	−0.396	0.918	0.670	0.026
Omega 9 (%)	−0.556	0.635	0.387	0.06	0.090	0.650	0.891	0.002
Hydroxytyrosol (ppm)	0.038	0.019	0.058	0.11	0.005	0.020	0.790	0.014
Tyrosol (ppm)	0.003	0.007	0.721	0.01	−0.007	0.004	0.081	0.221
Olive oil as oil most frequently used with								
CRP (ngmL)	−504.39	660.75	0.450	0.06	131.02	582.42	0.824	0.003
TNF-α (pgmL)	−0.13320	0.12563	0.296	0.048	−0.37219	0.15281	0.023	0.199
IL-6 (pgmL)	0.61535	0.83815	0.468	0.039	0.70396	0.92189	0.453	0.048
MUFA (%)	0.131	1.011	0.898	0.06	0.696	0.957	0.475	0.024
Oleic acid (%)	0.036	0.948	0.970	0.06	0.463	0.808	0.572	0.020
Omega 3 (%)	0.89628	0.36287	0.019	0.183	0.20528	0.42999	0.637	0.010
Omega 6 (%)	−1.33274	1.34777	0.330	0.027	−0.42694	1.37174	0.758	0.022
Omega 9 (%)	0.10684	1.03085	0.918	0.047	0.56453	0.96316	0.563	0.016
Hydroxytyrosol (ppm)	0.02982	0.03239	0.364	0.031	−0.05012	0.02876	0.097	0.141
Tyrosol (ppm)	−0.01744	0.01281	0.185	0.067	−0.00006872	0.00715	0.992	0.063
Fast Food with								
TNF-α (pgmL)	0.02919	0.05935	0.626	0.024	0.11427	0.08914	0.212	0.066
IL-6 (pgmL)	−0.16980	0.38495	0.662	0.028	−0.03013	0.50372	0.953	0.025
SFA (%)	0.25249	0.23367	0.287	0.124	−0.40098	0.38530	0.308	0.076
Trans fat (%)	0.02804	0.02014	0.173	0.069	−0.07291	0.11761	0.541	0.107
Red and processed meats with								
TNF-α (pgmL)	−0.01099	0.03673	0.766	0.020	0.06130	0.03965	0.135	0.092
IL-6 (pgmL)	0.10635	0.23932	0.660	0.028	−0.05615	0.22705	0.807	0.027
SFA (%)	0.16769	0.14391	0.252	0.129	−0.04400	0.17753	0.806	0.036
Omega 3 (%)	−0.22092	0.10696	0.046	0.145	−0.17799	0.09880	0.084	0.119
Omega 6 (%)	0.17853	0.39255	0.652	0.006	0.15885	0.33333	0.638	0.027
Nuts with								
TNF-α (pgmL)	0.00314	0.05098	0.951	0.018	0.05098	0.05688	0.379	0.034
IL-6 (pgmL)	−0.41093	0.31558	0.202	0.072	−0.13178	0.31502	0.679	0.031
Omega 3 (%)	0.16628	0.15453	0.289	0.071	0.03149	0.14626	0.831	0.002
Omega 6 (%)	−0.15244	0.54522	0.781	0.002	0.76669	0.43868	0.093	0.129
Linoleic acid (%)	0.02443	0.49517	0.961	0.004	0.97259	0.49793	0.063	0.172
n-6 Linolenic acid (%)	−0.03366	0.02793	0.236	0.041	−0.01286	0.03494	0.716	0.006
Fish with								
TNF-α (pgmL)	0.02446	0.04830	0.616	0.025	−0.07476	0.07311	0.317	0.043
IL-6 (pgmL)	−0.70823	0.29396	0.022	0.172	−0.00409	0.40835	0.992	0.024
Omega 3 (%)	0.27259	0.14209	0.063	0.132	0.17512	0.18567	0.355	0.036
Omega 6	−0.20296	0.51794	0.698	0.004	1.01657	0.56466	0.084	0.135
Alcoholic beverages with								
TNF-α (pgmL)	0.03047	0.15146	0.842	0.019	−0.09094	0.17645	0.611	0.012
IL-6 (pgmL)	−0.99832	0.98883	0.320	0.053	0.03948	0.97002	0.968	0.024
Hydroxytyrosol (ppm)	−0.02219	0.03874	0.571	0.017	−0.08268	0.02863	0.009	0.302
Tyrosol (ppm)	0.00420	0.01449	0.774	0.004	−0.00624	0.00872	0.484	0.090

Models adjusted for group assignment. CRP: C-Reactive Protein; MUFA: Monounsaturated fatty acids; TNF-α: Tumor necrosis factor; IL-6: Interleukin 6; SFA: Saturated Fatty Acids; mMDS: modified Mediterranean Diet Score.

**Table 5:** Spearman’s correlations of dietary fatty acid intakes with their corresponding plasma biomarkers at baseline and 6 months follow up in the pilot study of the “Feeding Americas Bravest.”

	Baseline Corresponding Plasma Biomarker				6-Months Follow up Corresponding Plasma Biomarker			
	Control		Intervention		Control Intervention		Intervention Self-Sustained Phase	
Nutrients (from FFQ)		*p*-Value		*p*-Value		*p*-Value		*p*-Value
SFA	0.137	0.524	0.178	0.406	−0.038	0.874	0.295	0.194
Lauric fatty acid	−0.094	0.662	0.227	0.286	−0.191	0.420	−0.054	0.818
Myristic fatty acid	−0.123	0.566	0.166	0.439	−0.315	0.177	0.184	0.425
Palmitic fatty acid	−0.096	0.654	0.133	0.537	0.177	0.455	0.570	0.007
Stearic fatty acid	0.020	0.925	−0.072	0.738	0.387	0.092	−0.128	0.581
Palmitoleic acid	−0.039	0.857	0.018	0.935	−0.614	0.004	0.226	0.324
MUFA	0.079	0.715	0.152	0.477	−0.295	0.206	0.262	0.251
Oleic acid	−0.165	0.441	−0.357	0.087	0.062	0.795	0.021	0.928
PUFA	−0.260	0.220	−0.059	0.785	0.134	0.573	−0.316	0.163
Linoleic acid	−0.191	0.373	0.210	0.324	0.127	0.593	−0.090	0.699
Alfa-Linolenic acid	0.217	0.307	0.302	0.152	0.073	0.760	−0.083	0.722
Omega-3 fatty acids	0.624	0.001	0.381	0.066	0.741	<0.001	0.396	0.075
Eicosapentaenoic fatty acid (EPA)	0.466	0.022	0.441	0.031	0.688	<0.001	0.621	0.003
Docosahexahenoico (DHA)	0.673	<0.001	0.347	0.097	0.775	<0.001	0.292	0.198
Total Trans fatty acid	0.080	0.711	−0.033	0.878	−0.081	0.733	−0.059	0.800
Conjugated linoleic acid	−0.148	0.490	0.122	0.570	−0.066	0.783	0.726	<0.001

SFA: Saturated Fatty Acids; MUFA: Monounsaturated fatty acids; PUFA: polyunsaturated fatty acids.

## Supplementary Materials

The following are available online at https://www.mdpi.com/2072-6643/11/9/2250/s1, Table S1: Modified Mediterranean Score, Table S2: Correlation between the scores.
