# Supplementary material for: Assessing Validity of Self-Reported Dietary Intake within a Mediterranean Diet Cluster Randomized Controlled Trial among US Firefighters
Source: Nutrients. 2019 Sep 19;11(9):2250. doi: 10.3390/nu11092250 (PMC6769698; doi:10.3390/nu11092250)
Supplement: Supplementary file 1 [file nutrients-11-02250-s001.pdf]

Supplementary Table S1. Modified Mediterranean Score.

Dietary Domain	Question(s)	Score Criteria	Score
Fast food	How many times per week do you eat fast-food or take-out food?	Never	4
		1 or less	3
		2-4	2
		5-7	1
		8+	0
Fruits	How many servings of fruit do you consume per day?	5+	4
		3-4	3
		2-3	2
		1 or less	1
		0	0
Vegetables	How many servings of vegetables (not including potatoes) do you consume per day?	5+	4
		3-4	3
		2-3	2
		1 or less	1
		0	0
Legumes	How many servings of legumes (e.g. beans, chickpeas, lentils) do you consume per week?	7+	4
		5-6	3
		3-4	2
		2-3	1
		1 or less	0
Nuts	How many servings of nuts (e.g. walnuts, almonds, hazelnuts, pistachio, peanuts) do you consume per week?	7+	4
		5-6	3
		3-4	2
		2-3	1
		1 or less	0
Sweet desserts	How many times per week do you eat sweet desserts (cake, cookies, pie, ice cream, etc.)?	1 or less	4
		2-3	3
		3-4	2
		5-6	1
		7+	0
Oil or fat at home	Which oil or fat do you use most often for cooking and serving food at home?	EVOO	5
		Olive oil	4
		Benechol or Smart Balance	3
		Corn or vegetable oil	2
		Margarine	1
		Butter, Lard or other animal fat, or None	0
Oil or fat at the firehouse	Which oil or fat do you use most often for cooking and serving food at the firehouse?	EVOO	5
		Olive oil	4
		Benechol or Smart Balance	3
		Corn or vegetable oil	2
		Margarine	1
		Butter, Lard or other animal fat, or None	0
Fried foods	How many times per week do you eat fried foods (french fries, fried chicken, chicken nuggets, etc.)?	Never	4
		1 or less	3
		2-3	2
		3-4	1
		5+	0
Breads or starches at home	Which bread or starch do you most frequently eat at home?	Whole wheat/multi-grain/pasta or brown rice	4
		I do not eat bread or starch / Durum wheat bread or dry pasta	3
		French/Italian/Multigrain or other crusty bread	2
		White bread/ macaroni/pasta/rice or potatoes, filled pasta	0

Breads or starches at the firehouse	Which bread or starch do you most frequently eat at the firehouse?	Whole wheat/multi-grain/pasta or brown rice	4
		I do not eat bread or starch / Durum wheat bread or dry pasta	3
		French/Italian/Multigrain or other crusty bread	2
		White bread/ macaroni/pasta/rice or potatoes, filled pasta	0
Ocean fish	How many times per week do you eat baked, broiled, grilled, or blackened (not fried) ocean fish (salmon, tuna, cod, haddock, etc.)?	5+	4
		3-4	3
		2-3	2
		1 or less	1
		0	0
Alcoholic beverages	How many alcoholic beverages (beer, wine, hard liquor, etc.) do you drink over a typical week?	5-20	4
		1-4	2
		21+	1
Beverages	When you drink alcoholic beverages, what type do you drink?	Red or White wine	2
		Beer / Hard liquors / Don't drink	0
Non-alcoholic beverages at home	Which of the following non-alcoholic beverages do you most frequently drink at home?	Water	4
		Juice / Tea / Coffee	2
		Milk / Fruit drink or punch / Diet cola/soda	1
		Cola/soda/Other	0
Non-alcoholic beverages at firehouse	Which of the following non-alcoholic beverages do you most frequently drink at the firehouse?	Water	4
		Juice / Tea / Coffee	2
		Milk / Fruit drink or punch / Diet cola/soda	1
		Cola/soda/Other	0

Supplementary Table S2. Correlation between the scores.

	mMDS	mMDS Derived from FFQ	PREDIMED
**Baseline**			
mMDS	1.0	0.74	0.72
mMDS Derived from FFQ	0.74	1.0	0.57
PREDIMED	0.72	0.57	1.0
**12 months**			
mMDS	1.0	0.42	0.69
mMDS Derived from FFQ	0.42	1.0	0.48
PREDIMED	0.69	0.48	1.0
**18 months**			
mMDS	1.0	0.59	0.58
mMDS Derived from FFQ	0.59	1.0	0.37
PREDIMED	0.58	0.37	1.0

mMDS: modified Mediterranean Diet; FFQ: Food Frequency Questionnaire
